# Effects of honey supplementation combined with different jumping exercise intensities on bone mass, serum bone metabolism markers and gonadotropins in female rats

**DOI:** 10.1186/1472-6882-14-126

**Published:** 2014-04-04

**Authors:** Maryam Mosavat, Foong Kiew Ooi, Mahaneem Mohamed

**Affiliations:** 1 Sport Science Unit, School of Medical Sciences, Universiti Sains Malaysia, Kubang Kerian, Malaysia; 2 Department of Physiology, School of Medical Sciences, Universiti Sains Malaysia, Kubang Kerian, Malaysia

**Keywords:** Bone, Female rats, Gonadotropins, Honey supplementation, Jumping exercise

## Abstract

**Background:**

The effects of high and low jumping exercise intensities combined with honey on bone and gonadotrophins were investigated in eighty four 9 week-old female rats.

**Methods:**

The experimental groups were 20 or 80 jumps per day combined with or without honey supplementation (HJ_20,_ HJ_80_, J_20_ and J_80_), honey supplementation (H), sedentary without supplementation control (C), and baseline control (C_0_) groups.

**Results:**

Study results showed that HJ_80_ elicited greatest beneficial effects on tibial and femoral mass, serum total calcium and alkaline phosphatase concentrations. There were significantly (p < 0.05) lower levels of serum follicle stimulating hormone concentrations in H, J_20_, J_80_ compared to C, with exception of HJ_20_ and HJ_80_. Serum luteinizing hormone concentrations were significantly (p < 0.05) greater in HJ_20_, HJ_80_ and J_20_ compared to J_80_.

**Conclusions:**

It appears that high intensity jumping exercise combined with honey supplementation resulted more discernable effects on bone. Meanwhile, honey may protect against the adverse effects induced by jumping exercise on gonadotropins in female rats.

## Background

It is well known that mechanical loading has an effective role in increasing bone health, in which an adequate exercise program can promote bone development and protect bone against age-related bone loss [1]. Evidences show that osteogenic effects of mechanical loading are dependent on the type, magnitude and rate of the applied load [2]. Dynamic and high magnitude loading such as jumping exercise which elicits great ground reaction force could elicit beneficial effects on bone health in rats [3-5] and humans [6-10]. Nevertheless, strenuous exercise is believed may elicit negative effects on bone properties [11-14].

Bone is a dynamic tissue that serves both mechanical and metabolic functions. Bone metabolism markers are generally accepted as good predictors of bone mass. Markers of bone formation and resorption reflect different stages of osteoblast proliferation, differentiation and bone resorption. In this study, tibial and femoral wet weights were measured to reflect bone mass, whereas serum total calcium was measured as one of the bone metabolism markers, serum alkaline phosphatase was measured as biomarker for bone formation, and C-terminal telopeptide of type 1 collagen (1CTP) was measured as biomarker of bone resorption.

Although substantial health benefits can be obtained through physical activity, high volume of exercise training is also associated with some serious risks such as menstrual disturbance in female athletes [15-18]. It is generally known that menstrual disturbance induced by strenuous physical activity may be due to hypothalamic dysfunctions. The relationship between the brain and the ovaries is called hypothalamic-pituitary-ovarian axis, this axis controls secretion of female reproductive hormones such as follicle-stimulating hormone (FSH) and luteinizing hormone (LH). It was mentioned by Mastorakos *et al.*[19] that strenuous exercise is related with a decreased hypothalamic-pituitary-adrenal secretion. The stress of exercise can stop the gonadal function, by the production of glucocorticoids and cathecholamines with activation of the corticotropin releasing hormone neurons. Additionally, it was mentioned by Warren & Perlroth [20] that low caloric input and high caloric expenditure can result in endocrine abnormalities, and this could be a factor which affects gonadotropin-releasing hormone suppression that manifests as menstrual disturbances with strenuous training. The consequence effects of strenuous exercise on female menstrual dysfunction consist of amenorrhea, infertility and osteoporosis.

Honey is a natural product with high concentrated solution of a complex mixture of sugars that has been widely used for its therapeutic effect. Honey contains about 200 substances such as sugar (fructose, glucose, maltose and sucrose), enzymes, flavonoids, minerals, organic acids, proteins, phenolic acids and vitamins [21]. Tualang honey which was used in the present study is a type of wild multiflora honey found in the Malaysian rain forest which contains phenolic compounds that possess relatively good antioxidant activity using 1,1-diphenyl-2-picrylhydrazil (DPPH) and ferric chloride antioxidant power (FRAP) assays, which is comparable with other reported honeys such as Romanian and Slovenian honeys [22]. Chepulis and Starkey [23] reported that honey feeding alone has shown its beneficial effects on bone mineral density in animals. Additionally, two previous studies have shown that honey has similar effects with hormone replacement therapy on bone densitometry [24] and prevents uterine atrophy in postmenopausal state [25]. Nevertheless, these previous studies did not report the underlying mechanisms that could explain the increase in bone mass by honey feeding.

Recently, it has been reported that honey supplementation of 1 g/kg body weight, 7 days/week combined with moderate jumping exercise intensity of 40 jumps/day, 5 days/week for 8 weeks may elicit beneficial effects on bone in young female rats [5,26]. Nevertheless, the effects of honey supplementation combined with other lower or higher jumping exercise intensities have not been investigated. A recent study done by Mohamed *et al.*[27] showed that honey significantly attenuated the toxic effects of cigarette smoke on spermatogenesis and testosterone level in male rats. Their study finding implies that honey might elicit protective effect against cigarette smoke-induced impaired testicular functions, and it may have a protective effects on disturbance of reproductive hormones in male rats. However, to date no study has been carried out to investigate whether combination of higher intensity of jumping exercise with honey may elicit different effects on bone health and gonadotropins compared to combination of lower intensity of jumping exercise with honey in female rats. Moreover, we hypothesize that honey as a source of energy and its antioxidant properties may protect against adverse effects induced by exercise on gonadotropin hormones. Therefore, the aim of this study was to investigate the effects of different jumping exercise intensities, i.e. low and high intensities combined with honey supplementation on bone parameters and gonadotropins in female rats.

## Methods

### Animals

Eighty four, 9-week old female Sprague–Dawley rats with initial body weight of 175–210 g were obtained from the Animal Research and Service Center, Universiti Sains Malaysia (USM). The experimental protocol was approved by Animal Ethics Committee, Universiti Sains Malaysia (USM/Animal Ethics Approval 2011/ [71][325]). Standard chow in pellet form (Gold Coin, Port Klang, Malaysia), and water were provided ad libitum to all rats in all groups throughout the study.

### Vaginal smear and body weight measurement

At the beginning and end of this study, vaginal smear was performed on diestrus phase for standardization to make sure that the rats started and ended the experiment at same hormonal phase. Vaginal secretion of each rat was collected with a plastic pipette filled with 10 μL of normal saline (NaCl 0.9%) by inserting the pipette tip into the rat vagina, then unstained materials were observed under a light microscope at 100 x magnification. Initial body weights were measured to the nearest 0.1 g by using a body weight weighing scale (Navigator^TM^, OHAUS Corporation, N2B110, Switzerland).

### Animal grouping

After initial body weight measurement and vaginal smear, the rats were block-randomised into 7 initial body-weigh-matched groups with 12 rats per each group: Baseline control that were sacrificed at 9 week-old at the beginning of the study (C_0_), sedentary group without supplementation and exercise with free cage activity for 8 weeks (C), low intensity jumping exercise with 20 jumps per day at 5 days per week for 8 weeks (J_20_), high intensity jumping exercise with 80 jumps per day for 5 days per week for 8 weeks (J_80_), honey supplementation for 7 days per week for 8 weeks (H), low intensity of 20 jumps per day for 5 days per week combined with honey supplementation for 8 weeks (HJ_20_), high intensity of 80 jumps per day for 5 days per week combined with honey supplementation for 8 weeks (HJ_80_).

### Honey supplementation

Honey supplementation was given to the rats with a dosage of 1 g per kg body weight per day by oral gavage force fed for 7 days per week. The prescribed honey dosage was based on Tavafzadeh [5]. The rats in combined honey with jumping exercise group (HJ_20_ & HJ_80_) were fed with honey, 30 minutes prior to the jumping exercise session. The dosage of honey was calculated based on the rat biweekly body weight [5].

### Training program

The training programme of this study was done 5 days per week according to the previous study [5], and consisted of 20 and 80 jumps per day for 8 weeks. Briefly, each rat in the jumping exercise groups was placed at the bottom of a specially designed wooden box, measuring 30.5 cm × 30.5 cm and 40 cm in length, width and height, respectively. The jumping exercise was initiated by applying an electrical current to the wired floor (electrical grid) of the box through a stimulator. When stimulated, each rat jumped from the floor of the box to catch the top edge of the box with its forepaws. The rat was then immediately returned to the floor of the box to repeat the procedure. The time required per jump was about 4 seconds. After a few days of training, the rats jumped without electrical stimulation. Rats in the sedentary groups were not given any electrical stimulus but to mimic the stress induced by handling, before and after jumping exercise, the sedentary rats were also just handled during the duration of the study.

### Blood collection and analysis

At the end of the experimental period, the rats were anaesthetised by lying for 2–3 minutes in a dried jar containing chloroform-soaked gauze pad, then they were decapitated by using a small guillotine (Scientific Research Instrument, UK). Blood from the decapitated site was collected into the 10 mL test-tube through a funnel, it was allowed to completely clotted and subsequently centrifuged at 4°C and 4000 RPM for 15 minutes (Health-Ratina 46RS, Germany). Serum was collected and stored under temperature of −80°C (Heto Ultra Freezer 3410, Denmark) for subsequent analysis of serum total calcium (Ca), serum alkaline phosphatase (ALP), serum C-terminal telopeptide of type 1 collagen (1CTP), serum follicle stimulating hormone (FSH) and serum luteinizing hormone (LH).

Levels of serum total calcium, serum alkaline phosphatase, serum C-terminal telopeptide type 1 collagen, serum follicle-stimulating hormone and serum luteinizing hormone were measured using commercial kits (Ca: Randox, UK; ALP: AMP Buffer, UK; 1CTP: Creative diagnostic ELISA Kit, USA; Rat FSH: Cusbio ELISA Kit, USA; LH: Cusbio ELISA Kit, USA).

The intra-assay coefficient of variation (CV) of the assay kits were 0.9%, 0.7% and 0.4% at 2.12, 2.29 and 3.32 mmol.l^−1^ of serum total calcium concentration respectively, and the interassay CV were 1.5%, 1.6% and 0.8% at 2.10, 2.28 and 3.28 mmol.l^−1^ of serum total calcium concentration respectively. For serum alkaline phosphatase, the intraassay coefficient of variation (CV) of the assay kits were 0.67%, 0.47%, 0.45% and 0.61% at 63.3, 84.1, 214.7 and 304.7 U/L ^-1^ of serum alkaline phosphatase concentration respectively, and the interassay CV were 0.67%, 0.27%, 0.47% and 0.67% at 62.0, 85.6, 210.6 and 298.3 U/L ^-1^ of serum alkaline phosphatase concentration respectively. For both serum follicle-stimulating hormone (FSH) and luteinizing hormone (LH), the intra- and inter assay precision within an assay was less than 15%. The detection range of the assay kits of serum 1CTP was 0.156-10 ng/mL, and the minimum detectable dose of serum 1CTP was less than 0.03 ng/mL.

### Bone measurements

The left hind legs (tibia and femur) of the rats were dissected and the fleshy tissues were cleaned from the tibia and femur. Bone mass; i.e. tibial and femoral wet weights were measured using an electronic balance (Denver Instrument Company, AA-160 USA) [5]. Subsequently, tibia and femur were then immersed in a mixture of chloroform and methanol solvent (2:1 by volume respectively) for one week to remove the fat from the bones. Following this, the bones were oven dried at 80ºC for 24 hours (Isuzu Model 2–2020, Isuzu Seisakusho Co., Ltd., Japan). After drying, the fat free dry weight (to the nearest 0.01 mg) was determined on an electronic balance (ER-180A, A&D Company, Japan). Bone fat free dry weight is an indicator of bone mass, where it refers to bone weight with only the solid composition, i.e. mineral and organic (matrix) phases of the bone.

### Statistical analysis

Statistical analysis was performed by using the Statistical Package of Social Sciences (SPSS) Version 18.0. The power of the study was set at 80% with 95% confidence interval. All the data are presented as mean [95% confidence interval]. After checking normality and homogeneity, the data with normal distribution and homogenous variances were analysed using one-way analysis of variance (*ANOVA)* to determine the significance of the difference between groups. Whilst the One-way *ANOVA* revealed a significant difference, *post hoc* (least significant differences test) was used to determine the differences between specific means. The *P* value of <0.05 was considered as statistically significant and used for all the comparisons.

## Results

The mean initial and final body weights, tibial and femoral wet weights are presented in Table 1. There was no significant difference in initial and final body weights of the rats among all the groups. This study also showed that tibial and femoral wet weights were significantly (p < 0.05) greater in H, J_20_, J_80_, HJ_20_ and HJ_80_ groups compared to C and C_0_, respectively_._ Additionally, there were significantly (p < 0.05) greater values of tibial and femoral wet weights in HJ_80_ compared to all the other groups. In tibial fat free dry weight, there was significantly (p < 0.05) greater value in J_80_ compared to J_20_, and HJ_80_ compared to HJ_20_ respectively. Meanwhile, statistically significant (p < 0.05) greater value of tibial fat free dry weight (TFFW) was exhibited in HJ_80_ compared to H. In femoral fat free dry weight, there were significantly (p < 0.05) greater values in H, J_80_ and HJ_80_ compared to C, and significantly (p < 0.05) greater femoral fat free dry weight value was observed in HJ_80_ compared to HJ_20_.

**Table 1:** Initial and final body weights, left tibial and femoral wet and fat free dry weights of the rats

**Groups**	**Initial body weight (g)**	**Final body weight (g)**	**Tibial wet weight (mg)**	**Tibial fat free dry weight (mg)**	**Femoral wet weight (mg)**	**Femoral fat free dry weight (mg)**
**C**_**0**_	-----	196.47[185.61,207.32]	0.52[0.50,0.55]	0.29[0.27,0.31]	0.66[0.63,0.71]	0.37[0.35,0.39]
**C**	186.74[176.22,197.25]	245.64[233.48, 257.81]	0.57[0.55,0.60]	0.39[0.37,0.41]^a^	0.78 [0.75,0.80]	0.52[0.49,0.54]^a^
**H**	192.54[182.19, 202.89]	255.37[241.12,269.62]	0.66[0.63,0.69]^a,b^	0.43[0.41,0.45]^a,b^	0.86[0.83,0.90]^a,b^	0.56[0.54,0.58]^a,b^
**J**_**20**_	188.93[182.23,195.63]	250.64[243.13,258.15]	0.65[0.63,0.67]^a,b^	0.42[0.40,044]^a,b^	0.87[0.84,0.90]^a,b^	0.55[0.53,0.57]^a^
**J**_**80**_	189.07[182.85,195.29]	250.37[238.30,262,44]	0.65[0.62,0.68]^a,b^	0.45[0.42,0.48]^a,b,d^	0.86[0.82,0.90]^a,b^	0.57[0.55,0.59]^a,b^
**HJ**_**20**_	191.63[182.90,20037]	246.75[231.71,261.78]	0.65[0.62,0.68]^a,b^	0.42[0.40,044]^a,b,e^	0.87[0.83,0.91]^a,b^	0.54[0.51,0.58]^a^
**HJ**_**80**_	193.33[187.90,198.80]	254.63[241.51,267.74]	0.70[0.68,0.72]^a,b,c,d,e,f^	0.45[0.41,0.49]^a,b,c,f,^	0.93[0.90,0.95]^a,b,c,d,e,f^	0.58[0.54,0.62]^a,b,f^

Values are mean [95%confidence interval]. Data were analysed by One-way *ANOVA.* Each group consisted of 12 rats. ^a^significantly different from C_0_ (p < 0.05); ^b^significantly different from C (p < 0.05); ^c^significantly different from H (p < 0.05); ^d^significantly different from J_20_ (p < 0.05); ^e^significantly different from J_80_ (p < 0.05); ^f^significantly different from HJ_20_ (p < 0.05). C_0:_ Baseline control; C: Sedentary without honey and jumping exercise; H: Honey 1 g/kg body weight/day; J_20:_ Low intensity of 20 jumps/ day; J_80_: High intensity of 80 jumps/ day; HJ_20:_ Low intensity of 20 jumps/ day combined with honey supplementation; HJ_80_: High intensity of 80 jumps/day combined with honey supplementation.

Mean serum total calcium (Ca), serum alkaline phosphatase (ALP) and serum C-terminal telopeptide type 1 collagen (1CTP) are presented in Table 2. Serum Ca levels were significantly (p < 0.05) higher in J_20_ and HJ_80_ compared to C and C_0_, respectively. There was significantly (p < 0.05) higher level of serum Ca in HJ_80_ compared to H. Serum ALP levels were significantly (p < 0.05) higher in HJ_80_ and H compared to C. Additionally, serum ALP levels were significantly (p < 0.05) higher in H, HJ_20_ and HJ_80_ compared to J_20_. Serum 1CTP levels were significantly (p < 0.05) lower in all the groups compared to C_0_, with exception of C. There were significantly (p < 0.05) lower values of serum 1CTP level in H, J_20_, J_80_, HJ_20_ and HJ_80_ compared to C. Additionally, levels of serum 1CTP were significantly (p < 0.05) lower in H, J_80_ and HJ_80_ compared to HJ_20_.

**Table 2:** Bone metabolism markers: serum total calcium (Ca), serum alkaline phosphatase (ALP) and serum C-terminal telopeptide of type 1 collagen (1CTP)

**Groups**	**Ca (mmol/L)**	**ALP (U/L)**	**1CTP (ng/ mL)**
**C**_**0**_	2.72[2.64,279]	184.67[156.44,212.90]	1.05[1.00,1.10]
**C**	2.72[2.64,2.79]	98.36[89.87,106.86]^a^	1.04[0.95, 1.1]
**H**	2.73 [2.65,2.81]	124.18[108.33,140.04]^a,b^	0.85[0.95,1.13]^a,b^
**J**_**20**_	2.78[2.62,2.94]	102.25[87.61,116.89]^a^	0.89[0.87,0.92]^a,b^
**J**_**80**_	2.79[2.71,2.87]	112.10[108.33,140.04]^a^	0.86[0.82,0.90]^a,b^
**HJ**_**20**_	2.78[2.67,2.90]	120.55[108.55,132.54] ^a,d^	0.95[0.87,1.04]^a,b,c,e^
**HJ**_**80**_	2.89[2.71,3.07]^a,b,c^	133.60[111.22,155.98]^a,b,d^	0.84[0.79,0.91]^a,b,f^

Values are mean [95%confidence interval]. Data were analysed by One-way *ANOVA.* Each group consisted of 12 rats. ^a^significantly different from C_0_ (p < 0.05); ^b^significantly different from C (p < 0.05); ^c^significantly different from H (p < 0.05); ^d^significantly different from J_20_ (p < 0.05); ^e^significantly different from J_80_ (p < 0.05); ^f^significantly different from HJ_20_ (p < 0.05). C_0:_ Baseline control; C: Sedentary without honey and jumping exercise; H: Honey 1 g/kg body weight/ day; J_20:_ Low intensity of 20 jumps/ day; J_80_: High intensity of 80 jumps/day; HJ_20:_ Low intensity of 20 jumps/ day combined with honey supplementation; HJ_80_: High intensity of 80 jumps/day combined with honey supplementation.

Mean serum follicle stimulating hormone (FSH) and serum luteinizing hormone (LH) concentrations of all the groups are shown in Table 3. Serum FSH was significantly (p < 0.05) lower in H group compared to C_0._ There were significantly (p < 0.05) lower values of serum FSH in H, J_20_, J_80_ compared to C, with exception of HJ_20_ and HJ_80_. Serum LH was significantly (p < 0.05) higher in all the experimental groups compared to C_0_, with exception of J_80._ Meanwhile_,_ LH levels were significantly (p < 0.05) lower in all the experimental groups compared to C, with exception of J_20_ and HJ_80_. There were significantly (p < 0.05) higher LH levels in J_20,_ HJ_20_ and HJ_80_ compared to J_80_.

**Table 3:** Serum follicle stimulating hormone (FSH) and serum luteinizing hormone (LH)

**Groups**	**FSH (mlU/mL)**	**LH (mlU/mL)**
**C**_**0**_	67.44[36.58,98.30]	0.67[0.53,0.82]
**C**	100.74[68.91,132.58]	1.90[1.35,2.46]^a^
**H**	48.52[17.84,79,19]^b^	1.23[0.97,1.48]^a,b^
**J**_**20**_	48.32[33.95,62.70]^b^	1.24[1.04,1.44]^a^
**J**_**80**_	50.32[21.01,79.63]^b^	0.76[0.60,0.93]^b,c,d^
**HJ**_**20**_	75.58[33.68,117,48]	1.06[0.83,1.28]^a,b,e^
**HJ**_**80**_	61.82[30.56,93.08]	1.41[0.97,1.85]^a,e^

Values are mean [95%confidence interval]. Data were analysed by One-way *ANOVA.* Each group consisted of 12 rats. ^a^significantly different from C_0_ (p < 0.05); ^b^significantly different from C (p < 0.05); ^c^significantly different from H (p < 0.05); ^d^significantly different from J_20_ (p < 0.05); ^e^significantly different from J_80_ (p < 0.05). C_0:_ Baseline control; C: Sedentary without honey and jumping exercise; H: Honey 1 g/kg body weight/day; J_20:_ Low intensity of 20 jumps/day; J_80_: High intensity of 80 jumps/day; HJ_20:_ Low intensity of 20 jumps/day combined with honey supplementation; HJ_80_: High intensity of 80 jumps/day combined with honey supplementation.

## Discussion

The present study found that training regimen of high intensity jumping exercise containing 80 jumps per day combined with 1 g per kg body weight of daily honey supplementation elicited more discernable beneficial effects on tibial and femoral mass compared to low intensity jumping exercise containing 20 jumps per day combined with honey supplementation, jumping exercise alone and honey supplementation alone in young female rats. We observed that bone wet and fat free dry weights as indicators of bone mass with both organic and mineral phases in high intensity jumping combined with honey supplementation group was significantly greater than all the other experimental groups. Meanwhile honey supplementation alone, and both low and high intensity jumping exercises with or without honey supplementation elicited beneficial effects on enhancing bone mass compared to sedentary without honey supplementation control rats. The present study also showed that high intensity jumping exercise combined with honey may elicit more beneficial effects on tibial mass compared to low intensity jumping exercise combined with honey supplementation.

The osteogenic effects of mechanical loading have been manifested by many studies. Loading is known as an essential factor in the bone formation [28]. Forwood [29] has suggested that mechanical loads greater than common force which met by skeleton can increase bone mass as result of increasing bone formation and reduction of bone turnover. It has been proven by animal studies that jump exercise as a high impact loading exercise model gives large ground reaction and muscular contraction forces to lower limb bones during landing [3,30]. Similarly, the present study finding was consistent with previous jumping exercise studies which reported positive effects of this type of exercise on bone mass in rats [3,5]. It is postulated that the rhythmic nature of dynamic loading in this type of high impact loading exercise may increase delivered blood volume to working muscle compared to low impact loading exercise [31], therefore beneficial effects on bone mass was observed in the present study. It was reported in a previous study that bone mass increased significantly with only 5 or 10 jumps per day, and high amount of loadings per day was not necessarily for increasing bone health [3]. Two other human studies also showed the efficiency of high-impact and low-repetition exercises on bone mineral density [32,33]. Our results are in agreement with these aforementioned previous studies with the evidences that jumping exercise as low as 20 jumps per day at 5 days per week for 8 weeks may have potential to increase bone mass in young rats.

Regarding the present positive finding of honey supplementation on bone mass, it was reported that ingestion of carbohydrates is effective in increasing calcium absorption and suppressing bone resorption in human [34] and animal [35], implying that carbohydrate ingestion may enhance bone mineralization and organic matrix reflected by increased bone mass, as observed in the present study among honey fed rats. In a previous study, Ariefdjohan and colleagues [36] have shown that acute feeding of honey could increase calcium absorption in rats. Therefore the finding of large increased in bone mass as shown by the great increased bone wet weight in rats with combination of honey supplementation and high intensity of 80 jumps per day in this study might be due to increased calcium absorption with honey supplementation. The present finding may reflect that high intensity jumping exercise with 80 jumps per day combined with honey supplementation could cause enhancement of blood flow to working muscle for supplying nutrients contained in honey such as calcium, phosphorus, magnesium and vitamins to the bone during exercise.

Regarding bone metabolism markers, it was found that serum total calcium was significantly higher in high intensity jumping combined with honey supplementation than honey alone group. This observation implies that 80 jumps per day combined with honey feeding were most effective in enhancing serum total calcium level in the rats. It is speculated that honey feeding and its carbohydrate constituents such as glucose, fructose, and raffinose enhanced calcium absorption from intestinal tract [36] through the large volume of blood delivered to the muscle and subsequently to the bone by performing jumping exercise [31].

Serum alkaline phosphatase, a bone formation marker, was significantly greater in high exercise intensity combined with honey group and honey supplementation alone group compared to sedentary without honey supplementation controls. Additionally, serum alkaline phosphatase was significantly higher in low and high intensity jumping exercise combined with honey supplementation group and honey supplementation group compared to low intensity jumping exercise alone group. It seems that jumping exercise in both high and low levels of intensity combined with honey supplementation may elicit beneficial effects on bone by increasing level of bone formation marker, nevertheless, more discernable effect was observed in combined 80 jumps per day with honey group. The positive finding in combined 80 jumps per day with honey group in serum formation marker was consistent with serum total calcium mentioned earlier. In a recent human study carried out by the present researcher, it was also observed that aerobic dance exercise combined with honey supplementation could increase level of bone formation marker in young woman [37]. Interestingly, the present study also found that honey supplementation alone was effective in increasing serum alkaline phosphatase, this observation may reflect that the vital components contained in honey such as sugar, i.e. fructose, glucose, maltose and sucrose; enzymes, flavonoids, minerals, oxidants, organic acids, proteins, phenolic acids and vitamins [21] may have played their roles in enhancing bone formation.

In bone resorption marker, it was evidenced in the present study that serum 1CTP levels were significantly lower in honey alone group, low and high intensity jumping exercise alone groups and combined 20 and 80 jumps per day with honey group when compared to sedentary without honey supplementation control group. Nevertheless, combined 80 jumps per day with honey group elicited more discernable effect on reducing the level of serum bone resorption marker than 20 jumps per day combined with honey supplementation.

The beneficial effects of honey alone on bone resorption revealed that the vital components contained in honey such as carbohydrates, minerals, vitamins and flavonoids [21] may have potential in reducing bone resorption in the present study. Similar findings of reduction of bone resorption marker as results of exercise as observed in the present study has been reported by Kishimoto *et al.*[38] which found that 10 jumps per day for 5 days per week decreased level of serum 1CTP after 2 weeks of training in female college-aged non-athletes. We also found that combined 80 jumps per day with honey elicited discernable effect on reducing the level of serum 1CTP. Nevertheless, a recent human study done by Ooi *et al.*[37] found that aerobic dance exercise consisted of 1 hour per day, 3 times per week combined with 20 g of Gelam honey for 7 days per week for a duration of 6 weeks did not show any significant effect on serum C-terminal telopeptide of type 1 collagen level in young female. The discrepancy between the present study and the aforementioned previous study results maybe due to variation in type and duration of the physical trainings and kind of recruited subjects in both studies. The reduction in bone resorption marker in combined exercise with honey group of the present study may be due to the effect of jumping exercise on enhancement of blood volume and osteoblastic activity, and there was suppression of parathyroid hormone-vitamin D mechanism subsequent of increased calcium absorption after honey feeding [39]. Additionally, it was reported by previous studies that kaempferol, a flavonoid, has osteogenic and anti-osteoclastogenic effects on rats [40,41]. Flavonoid is effective in bone loss reduction by increase the numbers of osteoprogenitor cells and inhibit osteoclastic activity [41]. Therefore, the significant reduction in bone resorption marker in combined honey with jumping exercise group may be due to increase in supply of flavonoids component of Tualang honey, i.e. kaempferol [42] through the increased of blood flow to the working muscle induced by jumping exercise.

In the present study, levels of FSH were lower in low and high intensity jumping exercise alone groups and honey supplementation alone group compared to sedentary without honey supplementation control group. However, there were no significant differences in both combined low and high intensity jumping with honey groups compared to sedentary without honey supplementation control group. These findings imply that honey combined with low and high intensity jumping exercise can maintain FSH level as sedentary without honey supplementation group. In a human study done by Valentino *et al*. [18], it was reported that intense ballet training was accompanied with lower level of reproductive hormone, i.e. FSH in young women. Meanwhile, it was reported by Williams and colleagues [43] that low energy availability and negative energy balance, i.e. dietary energy intake minus exercise energy expenditure following vigorous and regular exercise regimen reduces gonadotrophins secretion from anterior pituitary gland via hypothalamic-pituitary pathway. They have shown that calories supplementation which contained one quarter piece of fresh fruit, i.e. 25 kcal that was equal to 138–181% of calorie intake, during amenorrhea induced by strenuous exercise, caused increases in reproductive hormone levels, i.e. FSH and LH, and reestablished ovulatory cycles in female cynomolgus monkeys. Their finding confirmed that exercise-induced suppression of reproductive function is caused by the energy expenditure associated with vigorous regular exercise.

Our data revealed that honey supplementation alone was accompanied with low level of FSH. Similarly, Zaid *et al.*[25] reported that administration of low dose of 0.2 g per kg, medium dose of 1.0 g per kg and high dose of 2.0 g per kg Tualang honey were accompanied with nonsignificant lower level of serum FSH in honey treatment groups compared to ovariectomised control rats. The present study found that both low and high intensities of jumping exercise may have elicited negative effects on FSH level and the combination of honey supplementation and exercise in both levels of intensities could improve the adverse effects induced by jumping exercise on FSH level.

In the present study, LH level was significantly lower in all the experimental groups compared to sedentary without supplementation control group, with exception of higher intensity jumping exercise combined with honey and lower intensity jumping exercise. Moreover, level of LH were significantly higher in both higher and lower intensities jumping combined with honey and lower intensity jumping compared to high intensity jumping without honey supplementation. As we explained before, exercise especially high intense exercise can affect release of the amount of hypothalamic hormone and reduction in the level of gonadotrophins, i.e. LH [44,45]. It was also reported that restricted energy avaibility due to intense exercise via disruption in gonadotropin releasing hormone can cause reduction in LH concentration in female [43,46]. In the present study, lower level of serum LH in the rats of honey supplementation group compared to sedentary without supplementation group may be implies that rats in the honey group were under stress due to force feeding and honey alone did not have protective effects on luteinizing hormone. Nevertheless, the precise mechanism of lower level of LH in HJ_20_ group compared to sedentary group is unknown and further investigation is needed. The present finding of lower level of serum LH in the J_80_ compared to J_20_, HJ_20_ and HJ_80_ implied that high intensity jumping exercise without honey supplementation may have elicited negative effects on serum LH. This result implies that honey, a high concentrated source of energy with 313 calories per 100 g and antioxidants such as flavonoids and phenolic acid [24] may have potential to elicit beneficial effects on reduction of adverse effect induced by high intensity jumping exercise.

Body composition such as fat or muscle mass are potent stimulators of bone mass. One of the limitations of the present study was that fat mass was not measured, and the lower limb muscle of the rats were not collected and weighed for adjusting the bone mass value of the rats. The present finding may reflect the potential use of honey for maintaining bone health and reproductive functions. However, it is suggested to monitor insulin sensitivity for detecting the possible metabolic side effects due to an overload of sugar amount for those who are supplemented with honey in their daily life. Nevertheless, further studies are needed to elucidate the exact mechanism of honey in improving bone metabolism and reproductive hormone levels.

## Conclusion

In conclusion, high intensity jumping exercise combined with honey may elicit beneficial effects on bone mass and bone metabolism markers. In addition, honey could elicit protective effects on disturbance of reproductive hormone levels induced by high and low intensities of jumping exercise. Therefore, honey may be able to be recommended to female athletes for maintaining their bone health and normal reproductive functions.

## Competing interests

The authors declare that they have no competing interests.

## Authors’ contributions

MM participated in data acquisition, data analysis and interpretation, writing of this manuscript. OFK, participated in the experimental design, data interpretation, editing and submission of this manuscript. MM, contributed in the experimental design, data interpretation and editing of the manuscript. All authors read and approved the final manuscript.

## Pre-publication history

The pre-publication history for this paper can be accessed here:

http://www.biomedcentral.com/1472-6882/14/126/prepub
